# "The Hospital" Medical Book Supplement.—No. IV

**Published:** 1908-02-22

**Authors:** 


### "THE HOSPITAL" MEDICAL BOOK SUPPLEMENT.—No. IV.

CONTENTS.

**The Ideal Text-Book** ... 555

**Medicine—**

Minor Medicine: A Treatise on the Nature and Treatment of Common Ailments ... 556

On Acute Pneumonia: Its Signs, Symptoms, and Treatment 556

Marine Climates in the Treatment of Tuberculosis ... 556

**Surgery—**

Prostatic Enlargement ... 556

Diseases of the Nose ... 557

Abdominal Hernia: Its Diagnosis and Treatment ... 557

**Ophthalmology—**

Diseases of the Eye: A Manual for Students and Practitioners 557

**Bacteriology—**

Lessons in Disinfection and Sterilisation ... 558

**Stray Notes—**

A Student's Book—Various Items—Some New Books of the Month ... 55

### THE IDEAL TEXT-BOOK.

In these days when publishers' catalogues have grown into moderate-sized volumes, and when the number of text-books has multiplied out of all proportion, possibly, to the demand, the choice of a text-book is not an easy matter. The individual needs of the reader will, of course, primarily influence the decision in any case. One man needs a work in some special branch—say operative surgery—and limits his selection to the dozen or more authoritative works on that subject. Another wishes to improve his knowledge of nervous diseases, and is similarly limited in his choice. But in both cases there are other factors which make him decide upon a particular work to the exclusion of others, and the decision is often arrived at without due regard to the factors which should weigh most with him in his selection.

The day of cheap reprints, so far as medical literature is concerned, has gone by. No standard text-book is cheap; and some are terribly expensive, because they do not satisfy. We need not stop to consider the subject of style, for few of us buy a medical or other professional book on account of its literary merits, and the number of well-written text-books in the English language may be counted on one's fingers. To a certain extent this absence of "readableness" in most of our text-books is an inevitable concomitant which we have to accept. Its cause is probably to be found in the fact that most of them are written by lecturers or clinical teachers who have not learned the value of condensation as the literary man understands it. To the professional writer condensation usually means a bald *précis* of facts, a schematic rendering of signs and symptoms, a dry and tedious enumeration of "things inside, things outside, and things in the wall of." The art of writing delightful short monographs on professional subjects is one which is not cultivated by our medical authors. They fatigue by overloading, by being intensely technical, and by wearisome repetition. It would be superfluous, therefore, to point out the deficiencies of "style" in most of our text-books. The few that stand out conspicuously by the care and attention that have been bestowed upon their sentences are the exceptions that go to prove the rule. They are so rare that when one meets with them one is apt to treasure them above their real value, simply because the beauty of their phrase, the wealth of their illustration, and the purity of their wording appear like oases in a desert of illiteracy and technicality.

But there are other faults in the ordinary text-book more easily amended than faults of style. The latter are due to the author, and their reformation can only be brought about by degrees. The former are the faults of the printer and the publisher, and can easily be remedied by paying a little more attention to the get-up of the book and making it conform to the type of the best text-book. It is a pity that our publishers have not all found out the value of using thick, unglazed paper, of large type for printing, and of limiting, as much as possible, the number of footnotes. Most of our best-known text-books are printed on highly-sized paper, which tries the eyesight, especially by electric light. Much of the eye-strain of which we hear so much is a result of bad printing and worse paper. The striking difference between the best American published text-books and those issued by English houses is obvious to anyone who has had the advantage of comparing two bulky volumes—the one printed and published on this side, and the other on the far side of the Atlantic.

The fact is the medical publisher, as much as the writer on medical subjects, has much to learn. It is true there has been a striking improvement lately. To instance only one sign of such advance—and we do so in no invidious sense—the recently published Oxford Medical Manuals are types which other publishers may take as models. The books which make up this series are excellently printed, on thick, not over-glazed, paper, and in general the subject-matter of each manual is freshly and interestingly written. The volumes are of a handy size, crown octavo, attractively bound, and well indexed. These may appear small points, but if properly attended to they go far to make or mar the popularity of a book. As a type of the other class—the class that is endured because their evils are looked upon as necessary, we may mention the older editions of the standard works on anatomy. What student has not shuddered at his anatomy text-book at times? To a great extent the antipathy which the subject inspires, which makes the student call it a dry science and an uninteresting study outside the dissecting-room, is due to the strain involved in perusing the closely printed pages, highly glazed on account of the illustrations.

The lesser faults to be found in our text-books are for the most part venial. Printers' errors, which to the uninitiated appear ghastly blunders, may be forgiven, for they are not usually puzzles to the reader. A graver fault is the absence or incompleteness of the index. The ideal text-book should have a complete index, arranged as to subjects and authors, and a bibliograhpy.

### MEDICINE.

Minor Medicine: A Treatise on the Nature and Treatment of Common Ailments. By W. E. Wynter, M.D., B.S. (Lond.), F.R.C.P., F.R.C.S., Physician to the Middlesex Hospital, etc. (London: Sidney Appleton. Small 8vo., pp. 275. Price 6s. net.)

This is a most excellent little volume, dealing solely with common, every-day ailments, many of which are entirely neglected in text-books of medicine—ailments which play a comparatively large part in general practice, but which cannot always be given scientific names. We agree with the author when he says that the change in the system of medical education some thirty years ago, whereby the custom of beginning as pupil to a medical man in general practice was abandoned in favour of proceeding straight from the course of general education to a medical school or university, has involved certain deficiencies in the knowledge of those so trained. Since the subjects of what might be regarded as trifling disorders either do not present themselves at a hospital or are intercepted in the casualty department in order to spare the time and energy of the visiting staff, the present-day student has little or no opportunity of familiarising himself with those slighter maladies which are likely to be among the first encountered when he begins practice. This is no doubt accentuated by the natural tendency of students to concentrate their attention on those organic diseases which are mostly inquired about at examinations, and to interest themselves in rare diseases, complex or extensive operations, and questions of higher research—matters of the utmost importance in regard to the progress of medicine, but with which those who are occupied in family practice, and who constitute perhaps ninety per cent. of the profession, are less directly concerned, at all events in early years. It was with the hope of conveying some information, and of arousing interest in the sphere of minor medicine, that the present volume was written. We think that the author has succeeded admirably; we wish examiners at the Colleges and elsewhere would ask candidates questions upon such subjects as are here dealt with—this would be the strongest stimulus to the student to study these small but important matters.

It is not possible to summarise all the contents of the book; but their nature will be clear when we mention the sort of things discussed:—Biliousness, flatulence, a "chill on the liver," heartburn, hiccough, cracked lips, pyorrhœa alveolaris, toothache, baldness stye, insect bites, harvest bugs, plant rashes, chilblains, whitlow, corns, bunions, blisters, relaxed throat, stitch in the side, cephalalgia, seasickness, palpitations, cramp, muscæ volitantes, ear-wax, ozœna, obesity, and so forth. The book is well printed on light, thick paper, so that it is pleasant both to read and to hold. Treatment of these simple affections is discussed at length, and in a very practical way. There are some very useful tables giving the dietary suitable to different ages, states of activity, and diseases, and there is a capital index. We feel certain that practitioners will read this volume both with pleasure and with profit, and we would recommend it to the student as well as to the qualified man.

On Acute Pneumonia, Its Signs, Symptoms, and Treatment. By Seymour Taylor, M.D., F.R.C.P., Physician to the West London Hospital, etc. (London: Henry J. Glaisher. Pp. 64. Price 1s. net.)

The general character of this publication itself is that of a reprint; the subject matter is, indeed, that of two lectures delivered at the Post-Graduate College, West London Hospital, and the author has published them, despite their imperfections, at the request of many members of his class. The second lecture, devoted entirely to treatment, is essentially practical, and it is the part of the pamphlet which will appeal to practitioners. The first lecture deals with the clinical aspects of pneumonia, in the ordinary way. There is nothing strikingly original in the whole. A recent publication by the author's colleague at the West London Hospital is, we think, decidedly more helpful in difficult cases, while for ordinary cases the practitioner will probably find that a good text-book article covers almost all the ground. As interesting clinical lectures, there is nothing to say against the publication before us, but as a separate work to keep upon one's bookshelf there is little to say in its favour.

Marine Climates in the Treatment of Tuberculosis. By William Ewart, M.D., F.R.C.P., Senior Physician to St. George's Hospital. Pp. 48. Not Illustrated. Small oblong. (Published by Messrs. Baillière, Tindall, and Cox, London. 1907. Price 1s. net.)

This little brochure is Dr. Ewart's opening address delivered at the Hastings meeting of the British Balneological and Climatological Society, on March 16, 1907. It is printed in nice large type, and the subject matter is well subdivided under headings at short intervals, so that it is very easy reading. There is nothing particularly original in it, but it is very useful to have an authoritative expression of opinion as to what sorts of tuberculous conditions are likely to benefit by treatment at the seaside, and what sorts are better sent to altitudes in the country. Dr. Ewart discusses the question from many standpoints, such as the temperament of the patient, and so forth. Broadly, he concludes that tuberculosis, other than pulmonary, is not yet treated as much as it should be at the seaside; that in regard to pulmonary tuberculosis, the best stages for seaside treatment are when it is only potential or suspected, rather than actually known to be present, or when it is known to be present but only a small area of lung is involved, and the trouble is not acute. In acute phthisis and in extensive phthisis the author advocates treatment in country altitudes rather than at the seaside.

### SURGERY.

Prostatic Enlargement. By Cuthbert S. Wallace, M.B., F.R.C.S. (London: Henry Frowde, Oxford University Press, and Hodder and Stoughton, Oxford Medical Manuals.)

This latest addition to the excellent "Oxford Medical Manuals" series is a good *résumé* of our knowledge of the condition with which it deals. The first chapter is devoted to a very clear and readable exposition of the surgical anatomy of the prostate gland. This chapter is well written and lucid. Attention is paid to detail, and some points not specially attended to in ordinary text-books of anatomy, yet of great importance to the surgeon, are fully dealt with. The second chapter deals with the experimental pathology and function of the genital glands. Mr. Wallace emphatically declares against vasectomy, mainly, as it seems, on the results of experimental operations upon animals. A review of the clinical evidence for and against the operation would have been, in our opinion, of far more service. Both vasectomy and Bier's operation of tying the internal iliac artery have been singularly neglected by writers on prostatic enlargement, and the results so far obtained are at least worthy of criticism. That either method can enter into competition with total or partial prostatectomy we do not for a moment attempt to argue; still, a summary of results and a brief discussion of the pros and cons would have been desirable in a work otherwise so complete as Mr. Wallace's volume. One of the most interesting chapters in the book deals with the bacteriology of prostatic enlargement. In view of the theory that the hypertrophy is the result of a catarrhal inflammation set up by bacteria which have gained entrance to the prostatic tissue from the urethra, it is interesting to find that the authors conclude that "while micro-organisms cause a certain amount of inflammation, which produces enlargement of the gland . . . bacterial infection is a secondary event," and again that "there is no evidence to support the view that enlargement . . . is of a gonorrhœal origin." In the sixth chapter Mr. Wallace discusses the prevailing views on the etiology of the condition. He rejects incontinently those suggesting that the enlargement is due to senile fibrosis, sexual excess, compensatory hypertrophy to counteract degenerative changes in the bladder, perverted testicular action and normal senile hypertrophy, discussing at greater length the three main theories of inflammatory, catarrhal, and neoplastic enlargement, and seemingly inclines to favour the last theory. The sections dealing with diagnosis, treatment, and operative interference are tersely and lucidly written, and present many points which are treated in a novel fashion, though in themselves not absolutely new. In the last chapter the question of carcinoma of the prostate is dealt with. The book is an able and thoughtful exposition of general and particular views; it can be recommended to all surgical students as a valuable essay on a subject not usually fully dealt with in the ordinary text-books. It shares with the other volumes in the series the advantages and attractions due to excellent printing and careful reading.

Diseases of the Nose. By Ernest B. Waggett, M.A., M.B., B.C. (London: Henry Frowde, Oxford University Press, and Hodder and Stoughton, Oxford Medical Manuals. Price 5s. net.)

Few students have an opportunity, during their hospital career, of getting a working knowledge of diseases of the nose. They acquire the experience in after life in private practice, sometimes at unnecessary trouble. Like most "specialities," rhinology is considered by the average practitioner as a difficult subject, and any uncommon disorder of the nose is approached by him with fear and trepidation and much searching of his manuals, which unfortunately supply him with little information. To the majority, therefore, Mr. Wagget's little work will prove highly useful. It gives a clear, though necessarily a condensed, account of the diseases of the nose and nasopharynx, of their treatment and of their diagnosis, and of the various operations devised for defects of the nose. The book is illustrated, a few instruments being figured, and several anatomical plates introduced.

Abdominal Hernia; its Diagnosis and Treatment. By W. B. de Garmo, M.D. (Philadelphia and London: Lippincott Company. Cloth; crown 8vo. 21s. net.)

There are several excellent treatises on hernia, but none of them has been written with special regard to the requirements of the general practitioner. They either cater for students or for operating surgeons, and are correspondingly either too elementary or too elaborate to serve the needs of the family physician. We therefore welcome this book, which is an attempt to deal with the subject from an entirely different point of view. The author, Professor de Garmo, whose work on hernia has made his name known not only in America but in this country as well, is especially fitted to write such a work that should appeal to the general practitioner, for he has had a large experience of "post-graduate" teaching, and the result of his attempt to bring certain salient points regarding the diagnosis and treatment before the notice of the reader in a vivid and informative fashion is singularly successful. While laying justifiable stress on the advisability of operating on every suitable case of hernia, Professor de Garmo points out that in many cases treatment by means of a carefully adjusted truss is often highly satisfactory. The chapter on trusses and their management is one that will be of real value to the practitioner, and it contains many valuable hints and "wrinkles" derived from the author's unusually wide experience. The illustrations, which have been judiciously chosen, everywhere tend to elucidate the text. The book is one of the best post-graduate manuals we have seen, and one which can be honestly recommended as a valuable addition to the practitioner's library. The details of operative work are given in a terse, clear, and wholly readable fashion, and the description of the technique of dealing with a strangulated hernia—technique with which every practitioner should be thoroughly familiar—is almost a model of what such descriptions should be. The author desired to write a work that would be of practical use to the "family physician," and he is to be congratulated on the success of his attempt. The book is well printed and strongly bound, the paper being free from that irritating gloss which is such a feature of books illustrated by photographic reproductions.

### OPHTHALMOLOGY.

Diseases of the Eye: A Manual for Students and Practitioners. By J. Herbert Parsons, M.B., B.S., F.R.C.S. (London. 1907. Pp. x. + 664. With numerous illustrations. Price 10s. 6d. net.)

It would seem to be one of the decrees of Fate that an ophthalmic surgeon must needs write a book on diseases of the eye. Some few, it is true, resist manfully and successfully, and to these "mute inglorious Miltons" a measure of gratitude is certainly due. For others, the stream of tendency is too strong, and they are swept into authorship. Little surprise then need be felt that Mr. Herbert Parsons has determined to number himself among those responsible for the issue of a manual on ophthalmology suitable for the wants of students and practitioners. He has already established a reputation as a writer in various departments of his subject, and hence it is not to be wondered at that he has yielded to the temptation which makes its appeal sooner or later to so many of, perhaps to all, his ophthalmic brethren. In his preface he endeavours to justify his consent on various grounds, and these are neither more nor less convincing than those to be found in other manuals produced in similar circumstances. Possibly it may be suggested that of books of this order there is already an ample supply. But the field is open and free, and Mr. Parsons has at least as much right as others to put in an appearance.

Concerning the merits of the book in relation to its declared purpose, there need be no hesitation in saying that the result is a decided success. Opportunity for originality, either in substance or in method, hardly exists in so well-trodden a pathway. But in following the well-established route Mr. Parsons shows himself to be a reliable, lucid, and helpful guide, and his book may be accepted by those for whom it is written with confidence and even with gratitude. Particularly is this true of the illustrations with which the book is generously and effectively supplied; the coloured illustrations of various diseased conditions of the fundus are highly successful. In the preliminary chapters an attempt is made to present the anatomical and physiological facts of the visual apparatus in so far as these bear upon clinical work, and both the attempt and the achievement merit recognittion. There is also a chapter on elementary physiological optics which is sufficient for all practical purposes, and may indeed be considered by some to be more severe than the necessities of the position demand. In a section termed "The Neurology of Vision," Mr. Parsons carries the reader along the intracranial portions of the visual pathway and discusses some of the clinical problems which are associated with lesions involving this part of the nervous system. The chapter is a useful but by no means an exhaustive one. In dealing with external diseases of the eye and with errors of refraction the book follows the usual lines, though it is by no means destitute of an individual note. Treatment secures a fair measure of recognition, and if at times the author seems somewhat dogmatic, this is infinitely better than a debate *pro* and *con* with uncertainty and confusion at the end of it. Altogether the book may be described as both useful and attractive, and it is worthy of high rank among its fellows.

### BACTERIOLOGY.

Lessons in Disinfection and Sterilisation. By F. W. Andrews, M.A., M.D., F.R.C.P., D.P.H., Lecturer on Pathology, Pathologist, and Sanitary Officer to St. Bartholomew's Hospital. (London: J. and A. Churchill. Pp. 222. Thirty-one illustrations. Second edition. Price 3s. 6d. net.)

This is an excellent little volume, written in a clear, readable style. It is free from complicated arguments, and it is intelligible even to the uninitiated. It is a pleasure, nowadays, to have a book before one which is free from crowds of references to the literature, but which none the less gives the important points that research work has brought to light. The book had its origin in a course of lectures to the nursing staff at St. Bartholomew's, in which the principles of disinfection and sterilisation of hands, instruments, bedding, and so forth had to be instilled into those who had no knowledge of bacteriology. The result, to judge by the book itself, is very good. The author has written for those who are not bacteriologists, but who require sufficient acquaintance with the principles and methods of bacteriology to be able to understand what they are doing when they attempt to carry out processes of disinfection. Every nurse and every person who is brought into contact with the sick-room, will from this book be able to gather a most useful, correct, and intelligible account of the manner of preventing contagion and of neutralising and destroying contagia. Not only nurses, and students, and lay helpers, but to an almost equal degree medical practitioners, will be able to derive greater benefit from this little book than they can from many other more extensive works, which are so often encumbered by a vast amount of detail not required by the general medical practitioner. The first few chapters discuss in a broad way the nature of bacteria, their classification, rate of multiplication, spore formation, and food; their relation to air, light, and temperature; and the general principles of their cultivation. Next follow chapters upon different modes of sterilisation—by heat in various forms, by chemicals of different kinds, and by filtration—with a full discussion of the conditions under which each kind of sterilisation may be of most use. Surgical cleanliness, the methods of dealing with the hands, with instruments, dressings, sponges, ligatures, and so forth, and the precautions necessary in midwifery cases, receive a chapter to themselves. Every midwife ought to possess a copy of this book, and act upon its instructions. Two chapters are devoted to disinfection in medical cases, detailing the precautions that nurses, lay helpers, and practitioners should observe inside and outside the sick-room in cases of all the infective diseases from measles to yellow fever—we are glad to see pneumonia in the list. The book ends with thirty pages which are of more particular interest to the practitioner, dealing as they do with such questions as the thermal death-points of non-sporing and of spore-forming bacteria, the relative germicidal powers of carbolic acid, formalin vapour, mercuric chloride, sulphurous acid gas, and so forth, the methods of testing the extent to which the disinfection of particular articles has been accomplished, and the disinfection of sputum. There is a serviceable index. Altogether we think the book admirably adapted to its purpose.

### STRAY NOTES.

**A Student's Book.** Messrs. Baillière, Tindall, and Cox's excellent "Aid" series is destined for the use of the student preparing for examination, and for such only. Regarded essentially as aids to examinations they are good manuals, and the fact that Mr. Joseph Cunnings' "Aids to Surgery" has so soon required a new edition is a proof of its popularity. The second edition of this little book is entirely commendable; the various sections are comprehensive though necessarily condensed. What it gives is up-to-date and useful. The price is 4s. net.

**Various Items.** The thirty-fifth annual issue of "Willing's Press Guide, 1908" (publishers James Willing, Junr., Limited, 125 Strand) is a full directory of the British and foreign press, and a useful reference book for those who have to do with the newspapers. "Town Planning In Theory and Practice" (published by the Garden City Association, 602 Birkbeck Bank Chambers, E.C., 1s. net) contains a fund of useful and interesting information, and deals with the problem of town planning from every standpoint, the public health, the ideal and the official included. "Disease. Its Prevention and Cause," by Dr. G. E. Richmond (H. K. Lewis, 136 Gower Street, W.C., 2s. net), is an essay whose main object is "to point out the very large number of diseases which are either spread by food or directly due to impurities in articles of diet." It is a sane exposition of certain dangers and a thoughtful discussion of some suggestions for amendment, but presents nothing strikingly new.

**Some New Books of the Month.** Messrs. J. and A. Churchill are about to publish the second and concluding volume of "The Labyrinth of Animals," by Dr. Albert A. Gray, aural surgeon to the Victoria Infirmary, Glasgow. The work is illustrated by numerous stereoscopic plates. Another new book to be published by the same firm is entitled "Abdominal Tuberculosis," by Mr. A. E. Maylard. The text will be copiously illustrated. New editions are nearly ready of the second of "Lectures on Medical Jurisprudence and Toxicology," by Dr. F. J. Smith, of the London Hospital. Additional lectures on "The Examination of the Person Alive and Dead," "Anæsthetics," and "Death Certification," have been included. Also the sixth edition of "A Simple Method of Water Analysis," by Dr. J. C. Thresh, of the London Hospital.

